# Intraoperative Hardware Failure of the Fassier–Duval Rescue System in a Pediatric Patient with Osteogenesis Imperfecta

**DOI:** 10.1155/2021/9982289

**Published:** 2021-05-25

**Authors:** Michael E. Kahan, Nathan R. Angerett, Jill C. Flanagan

**Affiliations:** ^1^Department of Orthopaedic Surgery, UPMC Pinnacle, Harrisburg, PA, USA; ^2^Department of Orthopaedics and Sports Medicine, Children's Hospital of Atlanta, Atlanta, GA, USA

## Abstract

The use of telescopic intramedullary rods for the treatment of lower extremity deformity in children with osteogenesis imperfecta has been well described. The reinforcement of these weakened weightbearing bones with intramedullary devices leads to improvements in mobility that progress into adulthood. Although the current telescopic intramedullary rod systems are an upgrade from earlier systems, they are still associated with high rates of reoperation and complication. We describe a unique complication encountered during a revision which involved the male retriever system for the Fassier–Duval rod (FDR) (Pega Medical, Quebec, CA) experienced intraoperatively. To our knowledge, this mechanism of failure has not been previously described in the literature.

## 1. Introduction

Sofield and Millar were the first to publish their experience with intramedullary rods in patients with osteogenesis imperfecta [1–4]. Since that time, the surgical treatment of osteogenesis imperfecta has significantly progressed. Currently, third-generation telescopic intramedullary nails are used to stabilize and prevent long bone fractures and to correct extremity deformity. The goals of treatment include the allowance of vertical mobilization, reduction in the number of fracture events, and prevention of deformity [5]. Improved nail designs have led to decreased rates of reoperation when compared to older systems [3]. However, as is true in many pediatric orthopedic surgical cases, planned revision surgery due to growth is exceedingly common. Regarding the FDR, the indications for revision surgery are many and include joint penetration, rod bending, proximal rod migration, and refracture [6, 7]. FDR removal is accomplished with the use of the FDR Rescue System 2.0 (Pega Medical, Quebec CA). The technique involves first placing the female rod retriever inside of the female rod and removing it. Next, the male rod retrieval shaft is placed over the male rod, the shaft is tightened, and then the male rod retrieval shaft and male rod are removed as one unit. We describe a case of failure and a unique salvage technique involving the FDR Rescue System 2.0 that occurred intraoperatively [8]. To our knowledge, no other cases involving fracture of the FDR male retrieval system or the described salvage technique have been described in the literature at this time. The patient provided informed consent for the publication of this case report.

## 2. Case History

**Figure 1:**
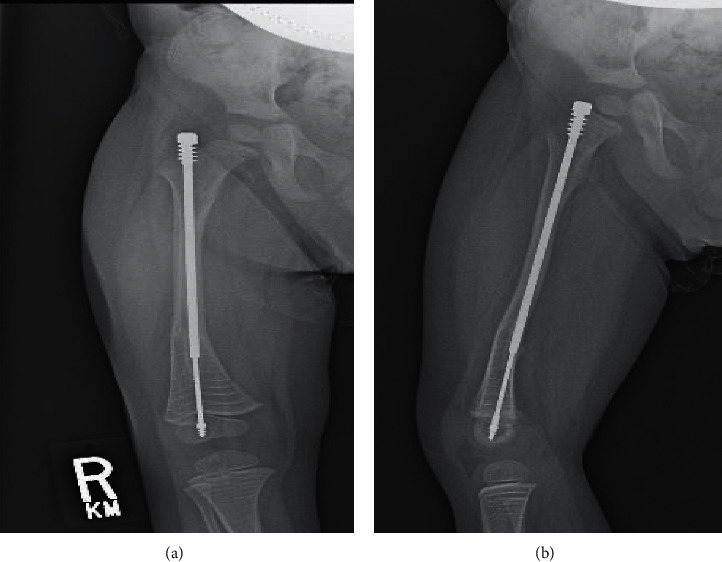
(a), (b) AP and lateral radiographs demonstrating the 3.2 mm Fassier–Duval rod in place with slight bending in the coronal plane.

3-year 6-month-old male with genetically diagnosed type III osteogenesis imperfecta presented with left thigh pain after a child fell onto his leg. Radiographs obtained at that time demonstrated a minimally displaced left femur fracture about a proximally migrated 3.2 mm FDR. The patient was initially made nonweightbearing and treated with immobilization in a posterior slab splint. Prior to the injury described above, there was an anticipation of a revision surgery of the bilateral FDRs at approximately 4 years of age due to proximal migration of the left FDR with recurrent femoral deformity and right-sided FDR bending. Given the recent left femur fracture and right-sided rod deformity as shown in Figure 1, the decision was made to expedite the surgery prior to the right-sided rod requiring an osteotomy to revise.

**Figure 2:**
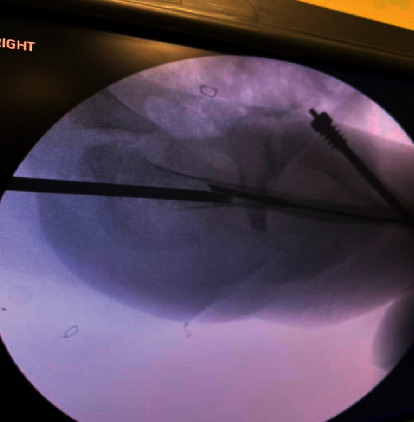
Lateral of the right femur demonstrating the fractured portion of the male retrieval shaft attached to the male rod.

**Figure 3:**
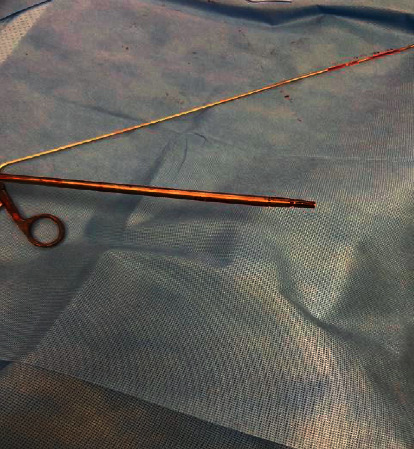
Gross picture of the 6.4 mm male rod retriever cannulated over the fractured 3.2 mm male rod retriever locking mechanism with a small portion protruding.

**Figure 4:**
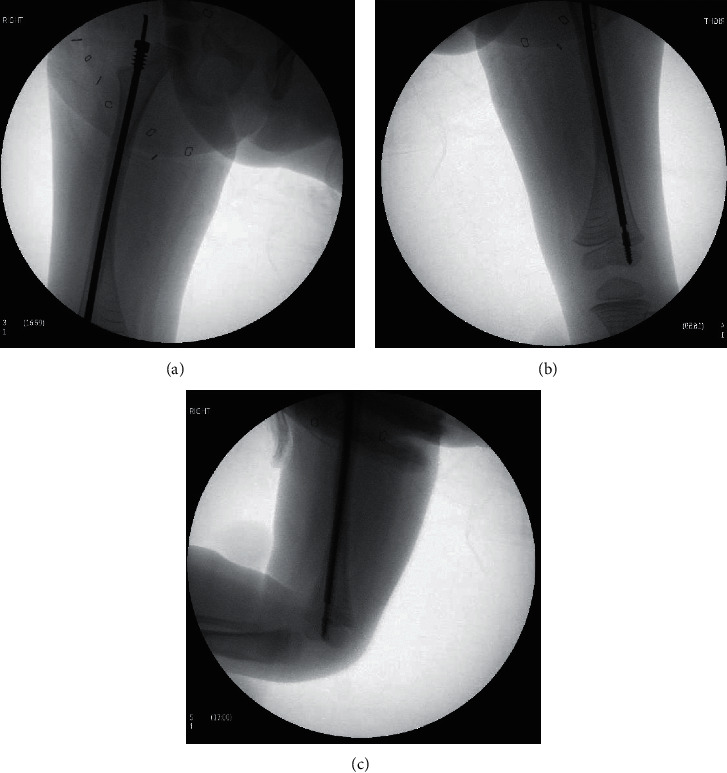
(a)–(c) AP and lateral radiographs of the 4 mm Fassier–Duval rod in place following removal of the fractured 3.2 mm male retrieval shaft and rod.

The patient underwent surgery at the age of 3 years and 8 months for revision of their bilateral Fassier–Duval rods. Regarding the left femur, the 3.2 mm FDR was successfully removed, a femoral osteotomy was performed to realign the femur, and a 4 mm FDR was successfully placed. Pertaining to the right femur, the female portion of the FDR was removed uneventfully followed by an unsuccessful attempt at removal of the male FDR. The male rod retriever shaft was placed over the male rod and tightened using the torque wrench. Following tightening, the male retriever was turned counterclockwise with a gentle pulling motion. At this point, the male retriever shaft fractured inside the femoral canal with the locking portion of the shaft still attached to the male rod as shown in Figure 2. Multiple unsuccessful attempts were made with different types of surgical instruments including micro and macropituitaries in an effort to engage the male retriever shaft fragment that was still attached to the male rod. Ultimately, a larger male retriever shaft (6.4 mm) was placed over the previously described shaft fragment and was able to be tightened around the fractured male retriever shaft to remove the fractured fragment and rod as one unit, as shown in Figure 3. Following the successful removal of the rod, a larger 4 mm FDR was placed in the appropriate position as shown in Figure 4. The patient had an uneventful postoperative course and was discharged from the hospital on postoperative day one.

## 3. Discussion

Patients with osteogenesis imperfecta suffer from brittle bones, joint hyperlaxity, and a number of different soft tissue defects [9]. Bisphosphonates remain the mainstay of medical treatment for these patients [10–12]. The orthopaedic surgical management utilizing intramedullary fixation to treat long bone deformity and prevent future fractures has been thoroughly described and has demonstrated successful long-term clinical and radiographic outcomes [1, 3, 5, 13]. Unfortunately, the intramedullary fixation of long bones in children with osteogenesis imperfecta has also demonstrated high rates of complication and reoperation [14].

Different telescopic intramedullary rodding systems have been developed, each with unique complications and technical difficulties. Jerosch et al. described complications including proximal rod migration/dislocation with the Bailey–Dubow Rod [14]. Nicolaou et al. described their experience with the Sheffield telescopic intramedullary rod system (Downs Surgical, Sheffield, United Kingdom) which included modifications from the Bailey–Dubow rod to include a larger fixed T-piece to prevent loosening within the epiphysis. However, this system was also associated with proximal rod migration and required a more invasive placement technique [2]. Regarding the FDR, which was used in our case, Azzam et al. described proximal rod migration rates of 16% which was nearly half the rate of proximal rod migration seen with the Bailey–Dubow rod [13]. This difference was felt to be attributed to improved fixation within the greater trochanter apophysis due to the threaded proximal portion of the female rod [15]. Other modes of FDR failure have been described including nail bending, joint penetration, and nonunion [3, 6, 13].

At this time, there have been no case reports that describe complications associated with the FDR Rescue System 2.0. In this case, the decision to revise the right femoral FDR was made due to mild rod deformation and to prevent impending femoral deformity. Preoperative radiographs demonstrate that the rod was not angled to a degree that would necessitate a femoral osteotomy to remove. Therefore, a standard rod removal technique was attempted using the FDR Rescue System to provide a minimally invasive surgery and significantly decrease recovery time. Intraoperative radiographs (Figure 2) demonstrate the fractured distal fragment of the male retriever shaft attached to the male rod which occurred during the removal attempt after the male retriever was successfully tightened on the rod. It is possible that due to the small intramedullary canal as well as the mild bend in the male rod, the male retrieval shaft became lodged in cortical bone leading to fracture of the locking mechanism at the time of removal. In this case, the male trephines were not used prior to rod removal as the retrieval shaft was able to be placed over the male rod without excessive force. Also, it is possible that an insufficient amount of the male rod was engulfed by the retrieval shaft leading to an excessive stress concentration at the junction between the male retriever shaft and the locking mechanism. Intraoperatively, we were able to use the 6.4 mm FDR retrieval shaft placed over the 3.2 mm fractured tightening system to remove the fractured piece and male rod as one unit (Figure 3). This involved minimal blood loss, and no femoral osteotomy was required. This is a novel technique not previously described.

Understanding the common causes of failure of these implants and their associated systems is important. Furthermore, knowledge of different techniques used to salvage such failures will decrease operative time and provide confidence when these complications are encountered intraoperatively.

## 4. Summary

The surgical treatment of patients with osteogenesis imperfecta is difficult and fraught with significant complications and revision surgery. However, it is often the only option to provide vertical ambulation and the ability to participate in physical therapy for these children. A thorough understanding of the intricacies involved with performing these types of surgery is paramount to decreasing complications. Additionally, an awareness of the mechanisms of implant failure and their different salvage techniques will lead to less invasive surgery and shorter operative times.
